# Preserved functional autonomic phenotype in adult mice overexpressing moderate levels of human alpha‐synuclein in oligodendrocytes

**DOI:** 10.14814/phy2.12209

**Published:** 2014-11-26

**Authors:** Jens Tank, Andrey C. da Costa‐Goncalves, Ilona Kamer, Fatimunnisa Qadri, Kiren Ubhi, Edward Rockenstein, André Diedrich, Eliezer Masliah, Volkmar Gross, Jens Jordan

**Affiliations:** 1 Institute of Clinical Pharmacology, Hannover Medical School, Hannover, Germany; 2 Max Delbrueck Center for Molecular Medicine, Berlin‐Buch, Germany; 3 Department of Neurosciences, University of California, San Diego, La Jolla, California, USA; 4 Department of Medicine, Division of Clinical Pharmacology, Autonomic Dysfunction Service, Vanderbilt University School of Medicine, Nashville, Tennessee, USA

**Keywords:** Autonomic failure, blood pressure, human alpha‐synuclein, multiple system atrophy, transgenic mice

## Abstract

Mice overexpressing human alpha‐synuclein in oligodendrocytes (MBP1‐*α*‐syn) recapitulate some key functional and neuropathological features of multiple system atrophy (MSA). Whether or not these mice develop severe autonomic failure, which is a key feature of human MSA, remains unknown. We explored cardiovascular autonomic regulation using long‐term blood pressure (BP) radiotelemetry and pharmacological testing. We instrumented 12 MBP1‐*α*‐syn mice and 11 wild‐type mice aged 9 months for radiotelemetry. Animals were tested with atropine, metoprolol, clonidine, and trimethaphan at 9 and 12 months age. We applied spectral and cross‐spectral analysis to assess heart rate (HR) and BP variability. At 9 months of age daytime BP (transgenic: 101 ± 2 vs. wild type: 99 ± 2 mmHg) and HR (497 ± 11 vs. 505 ± 16 beats/min) were similar. Circadian BP and HR rhythms were maintained. Nighttime BP (109 ± 2 vs. 108 ± 2 mmHg) and HR (575 ± 15 vs. 569 ± 14 beats/min), mean arterial BP responses to trimethaphan (−21 ± 8 vs. −10 ± 5 mmHg, *P* = 0.240) and to clonidine (−8 ± 3 vs. −5 ± 2 mmHg, *P* = 0.314) were similar. HR responses to atropine (+159 ± 24 vs. +146 ± 22 beats/min), and to clonidine (−188 ± 21 vs. −163 ± 33 beats/min) did not differ between strains. Baroreflex sensitivity (4 ± 1 vs. 4 ± 1 msec/mmHg) and HR variability (total power, 84 ± 17 vs. 65 ± 21 msec²) were similar under resting conditions and during pharmacological testing. Repeated measurements at 12 months of age provided similar results. In mice, moderate overexpression of human alpha‐synuclein in oligodendrocytes is not sufficient to induce overt autonomic failure. Additional mechanisms may be required to express the autonomic failure phenotype including higher levels of expression or more advanced age.

## Abstract (precis)

Mice overexpressing human alpha‐synuclein in oligodendrocytes recapitulate key functional and neuropathological features of multiple system atrophy (MSA). Whether or not these mice model MSA abnormalities in cardiovascular autonomic regulation is unknown. Therefore, we explored cardiovascular autonomic regulation using long‐term blood pressure radiotelemetry combined with detailed pharmacological testing in mice overexpressing moderate levels of human alpha‐synuclein under the control of the oligodendrocyte‐specific murine myelin basic protein promoter. The major finding was that the moderate overexpression of human alpha‐synuclein in oligodendrocytes was not sufficient to induce overt autonomic failure in this mouse model.

## Introduction

Multiple system atrophy (MSA) is a rare neurodegenerative disorder characterized by Parkinsonism poorly responding to levodopa, cerebellar ataxia, and autonomic failure in any combination (Kaufmann and Biaggioni 2003; Flabeau et al. 2010). Autonomic failure with severe orthostatic and postprandial hypotension is a disabling feature in many MSA patients (Kaufmann and Biaggioni 2003). MSA patients with autonomic failure are commonly hypertensive in the supine position such that the diurnal blood pressure profile is reversed. The peculiar clinical presentation results from complex abnormalities in sympathetic nervous system control through baroreflex mechanisms. Supine plasma norepinephrine concentrations and cardiac adrenergic terminals in thoracic 6‐[(18)F]fluorodopamine PET studies are within the normal range in MSA patients, suggesting that efferent sympathetic nerves are at least in part preserved (Sharabi et al. 2006). Therefore, pharmacological sympathetic inhibition lowers blood pressure more in MSA patients than in healthy subjects (Sharabi et al. 2006). However, sympathetic efferents are disconnected from central nervous system input without baroreflex feedback control. Symptomatic management of MSA‐associated autonomic failure remains disappointing and curative treatments do not exist. Identification of alpha‐synuclein containing glial cytoplasmic inclusions could provide a treatment target. Alpha‐synuclein, a 140 amino acid synaptic protein, is involved in the pathogenesis of MSA (Kaufmann and Biaggioni 2003; Ubhi et al. 2011). Neuropathologically, definite human MSA is characterized by the presence of misfolded alpha‐synuclein in oligodendroglial cells (Gilman et al. 2008). Transgenic and toxic animal models have been developed to assess candidate drugs for MSA treatment (Polinsky 1984; Robertson et al. 2001; Ubhi et al. 2008; Stefanova et al. 2009; Stemberger et al. 2011). Mice overexpressing human alpha‐synuclein in oligodendrocytes recapitulate key functional and neuropathological features of MSA (Shults et al. 2005; Stefanova et al. 2005; Rockenstein et al. 2007). Whether or not these mice model MSA abnormalities in cardiovascular autonomic regulation is unknown. Therefore, we explored cardiovascular autonomic regulation using long‐term blood pressure radiotelemetry combined with detailed pharmacological testing.

## Materials and Methods

### Animals

We obtained transgenic mice overexpressing human alpha‐synuclein under the control of the oligodendrocyte‐specific murine myelin basic protein promoter (MBP1‐*α*‐syn) from the Experimental Neuropath Laboratory, Department of Neurosciences, University of California, San Diego, La Jolla, CA. Mice had free access to standard chow (0.25% sodium, SNIFF Spezialitäten GmbH, Soest, Germany) and drinking water. They were maintained on a 12‐h light/12‐h dark cycle with light on at 6:00 am. The protocol was approved by the local council on animal care of the Max Delbrueck Center for Molecular Medicine, Berlin‐Buch, Germany, that corresponds to requirements of the American Physiological Society.

### Telemetry

We instrumented 12 MBP1‐*α*‐syn transgenic and 11 wild‐type mice aged 9 months for radiotelemetry (TA11PA‐C20, Data Sciences International, St. Paul, MN) to follow blood pressure and heart rate changes continuously over time. The telemetric techniques and the approaches employed to analyze autonomic cardiovascular regulation are described in detail elsewhere (Gross et al. 2008). Briefly, we anesthetized mice by isoflurane (CuraMed Pharma GmbH, Karlsruhe, Germany). Then, we advanced the pressure‐sensing catheter through the right femoral artery into the abdominal aorta and placed the transmitter in a subcutaneous pocket along the right flank. Mice recovered 10 days before baseline blood pressure and heart rate values were recorded. By this time, the mice had regained their circadian blood pressure and heart rate rhythm, and surgery and anesthesia‐induced changes in systolic blood pressure (SBP), diastolic blood pressure (DBP), mean arterial pressure (MAP), and HR had abated.

Data were sampled every 5 min for 10 sec continuously day and night with a sampling rate of 1000 Hz and stored on a hard disk. SBP, DBP, and HR were recorded using the DATAQUEST software (A.R.T. 2.1, Data Sciences International). Continuous beat‐by‐beat values of blood pressure and heart rate were recorded during morning hours for spectral analysis and pharmacological testing. Heart rate was computed from the pulse intervals of the blood pressure recordings.

### Pharmacological testing

At 9 and 12 months of age, we tested the same mice with atropine sulfate (2 mg/kg), metoprolol (4 mg/kg), clonidine (1 mg/kg), and trimethaphan (40 mg/kg). We dissolved all substances in 0.9% NaCl (10 *μ*l/g body weight) for intraperitoneal injection. Following injection, we returned mice to their cages and continued beat‐by‐beat blood pressure and heart rate recordings. Baseline values were obtained from 1 h recording before i.p. injections. The peak changes in BP and HR in response to each dose of vasoactive drugs were calculated by taking 10‐min average at the individual nadir response and expressing this as change relative to the 1‐h baseline prior to administration. The first minutes after returning the mice to the cage were excluded from analysis to avoid the influence of the initial stress. Beat‐by‐beat BP and HR values were derived from these 1‐h continuously recorded data (Gross et al. 2008). Mice recovered at least 24 h before we tested the next drug.

### Heart rate‐ and blood pressure variability

We applied spectral and cross‐spectral analysis to assess heart rate variability, blood pressure variability, and baroreflex sensitivity as described previously (Gross et al. 2008). Briefly, beat‐to‐beat values of detected pulse intervals and BP values were interpolated, low‐pass filtered (cutoff 6 Hz), and resampled at 12 Hz. We used 49‐sec data segments for spectral analysis. Linear trends were removed and power spectral density was estimated with the FFT‐based Welch algorithm using segments of 512 data points with 50% overlapping and Hanning window. The power in the frequency range of low frequencies (LF: 0.25–1.0 Hz) and high frequencies (HF: 1.0–6.0 Hz) were calculated. Five representative intervals were chosen for spectral analysis and averaged during 1‐h baseline and at maximum drug response. We calculated spontaneous baroreflex slope as the slope of the linear regression line between systolic BP and the subsequent pulse intervals using sequences defined as episodes of at least three heart beats associated with an increase in SBP per beat. Cross‐spectral baroreflex gain was defined as the mean magnitude value of transfer function between systolic BP and pulse interval in the low‐frequency band (BRS‐LF). The data analysis was performed with the PV‐wave software (Visual Numerics, Houston, TX).

### Histochemistry

Transgenic and wild‐type mice were sacrificed, whole brains were removed, and perfused overnight with 4% paraformaldehyde in PBS pH 7.4, then cryoprotected in 30% saccharose for 24–48 h at 4°C, until the brain sunk to bottom of the container. Brain sections (50 *μ*m) were cut on a cryostat at −20°C and transferred into cold phosphate‐buffered saline (PBS). The free‐floating brain sections were incubated in PBST (PBS‐0.5% Triton X‐100) for 30 min at room temperature, blocked with 10% normal donkey serum in PBST for 1 h at room temperature, and incubated in 1:200 rabbit polyclonal antihuman *α*‐synuclein antibody (Millipore‐Chemicon, Temecula, CA) at 4°C overnight. Sections were then rinsed with PBS, incubated with secondary antibodies, donkey anti‐rabbit Cy3 conjugated (Invitrogen, Carlsbad, CA) for 2 h at room temperature, and rinsed again in PBS. Free‐floating sections were then carefully placed on slides and mounted with Vectashield containing DAPI (Vector Labs. AXXORA, Lörrach, Germany) and imaged under Zeiss Axioplan microscope in order to verify the overexpression of human *α*‐synuclein. To locate and to count parasympathetic preganglionic motor neurons in the nucleus ambiguous serial vibratome sections from wild‐type (*n* = 6) and transgenic mice (*n* = 6, age 6 months) were immunolabeled with the rabbit polyclonal antibody against choline acetyltransferase (ChAT) and analyzed by the dissector method with the MBL serology system (Ubhi et al. 2013). Discrete groups of ChAT‐positive cells were identified and compared between strains.

### Statistics

Data are presented as mean ± SEM. Statistically significant differences in mean values were evaluated by one‐way ANOVA followed by Bonferroni post hoc test. For paired data, we used the nonparametric Wilcoxon signed‐rank test. A value of *P* < 0.05 was used to determine statistical significance**.**

## Results

**Figure 1.**
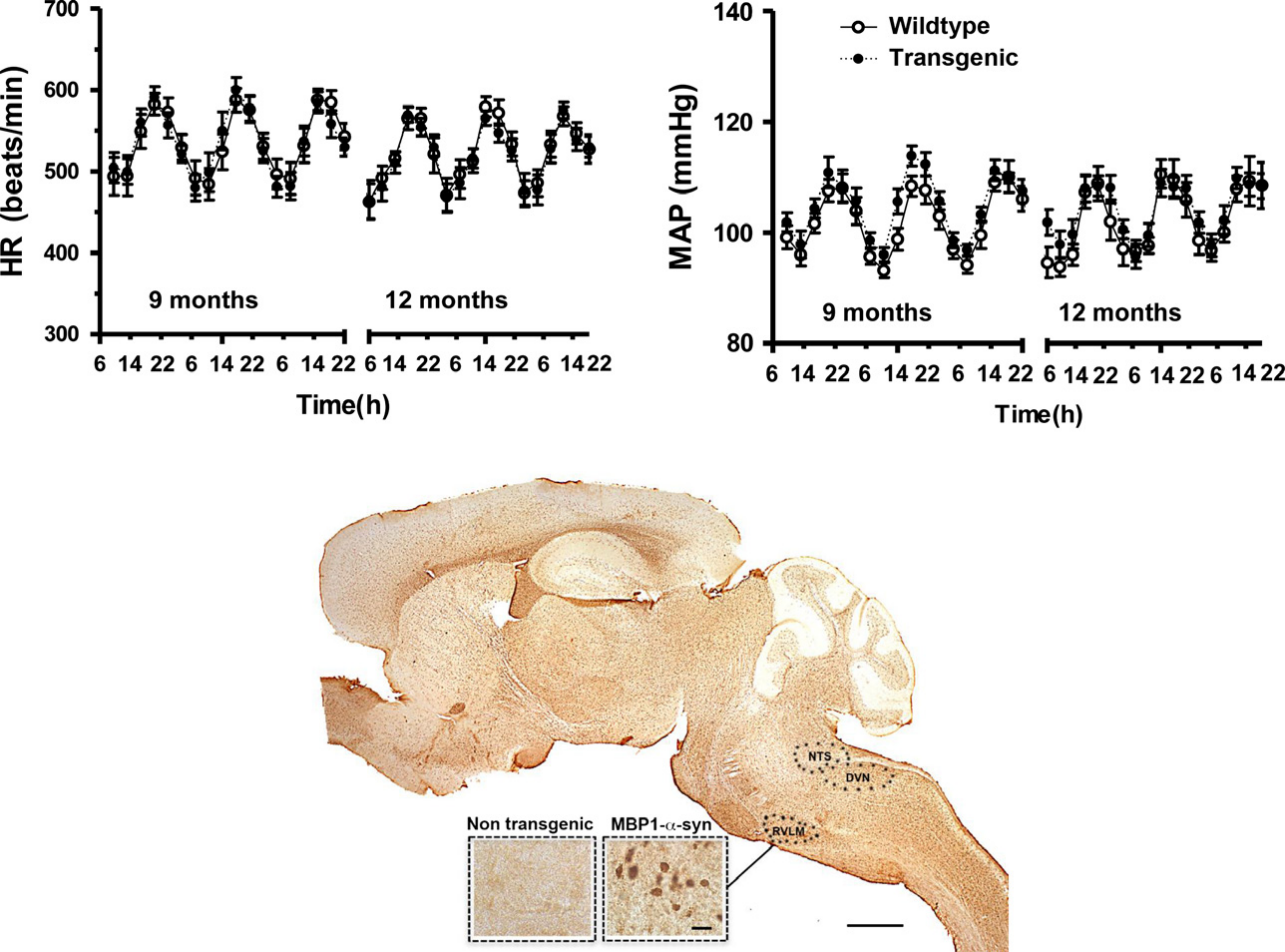
Circadian variations in heart rate (HR, top‐left) and mean arterial blood pressure (MAP, top‐right) measured over 3 days in MBP1‐*α*‐syn (tg) mice (transgenic) and in wild‐type mice (wild type) at 9 and 12 months of age. Distribution of alpha‐synuclein in MBP1‐*α*‐syn tg mice (bottom). The sagittal vibratome section is immunolabeled with an antibody against alpha‐synuclein (rabbit polyclonal from Millipore) illustrating the distribution of the alpha‐synuclein aggregates in oligodendroglial cells in various cortical and subcortical brain regions. Of the subcortical regions affected included those involved in hemodynamical regulation such as rostral ventrolateral medulla (RVLM), nucleus tractus solitarius (NTS), and dorsal vagus nucleus (DVN). The inset displays the alpha‐synuclein aggregates in oligodendroglial cells in the MBP1‐*α*‐syn mice as compared to a wild‐type control. Bar = 250 *µ*m for the low power image and 10 *µ*m for the inset.

At 9 months of age daytime blood pressure (tg: 101 ± 2 vs. wt: 99 ± 2 mmHg) and heart rate (tg: 497 ± 11 vs. wt: 505 ± 16 beats/min) were similar in MBP1‐*α*‐syn mice and in wild‐type mice. Circadian blood pressure and heart rate rhythms were maintained in MBP1‐*α*‐syn mice. Nighttime BP (tg: 109 ± 2 vs. wt: 108 ± 2 mmHg) and HR (tg: 575 ± 15 vs. wt: 569 ± 14 beats/min) were similar in both strains. Similar results were obtained at 12 months of age. Circadian variations in blood pressure and heart rate measured over 3 days for transgenic MBP1‐*α*‐syn and wild‐type mice at 9 and 12 months of age are illustrated in Figure 1 together with immunohistochemical staining of alpha‐synuclein in the brain of wild‐type and transgenic mice (bottom).

**Figure 2.**
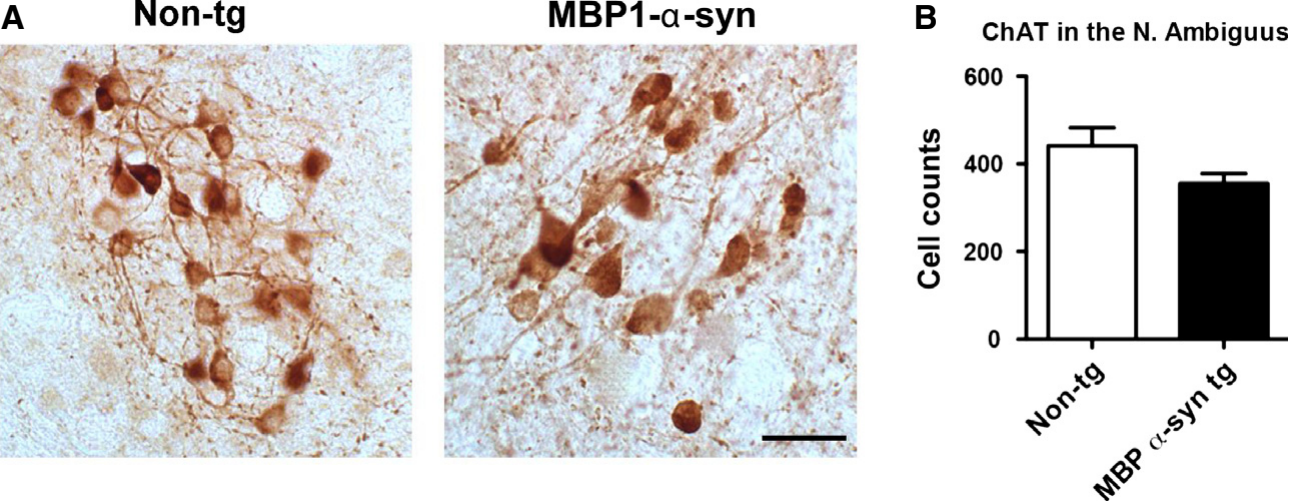
ChAT immunoreactivity in the nucleus ambiguus of nontransgenic and MBP1‐*α*‐syn tg mice (A). Serial vibratome sections from wild‐type (*n* = 6) and MBP1‐*α*‐syn tg mice (*n* = 6) were immunolabeled with the rabbit polyclonal antibody against ChAT and analyzed by the dissector method with the MBL serology system. Discrete groups of ChAT‐positive cells were identified, in both groups, compared to wild type the MBP1‐*α*‐syn tg mice showed a nonsignificant trend (B). Bar = 25 *µ*m.

**Figure 3.**
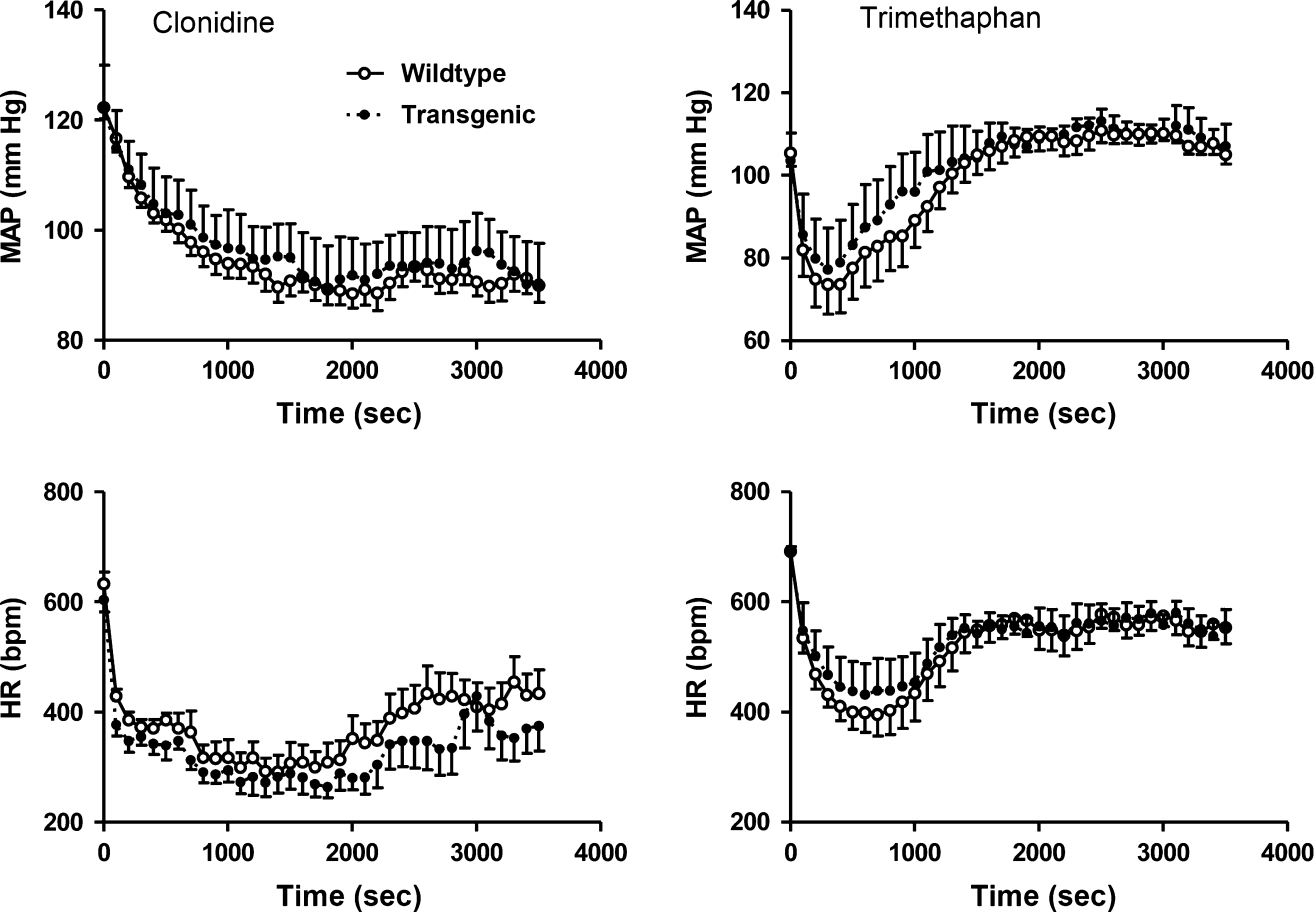
Heart rate (HR, bottom) and mean arterial pressure (MAP, top) after i.p. injection of 1 mg/kg clonidine (left) and after i.p. injection of 40 mg/kg trimethaphan (right) in MBP1‐*α*‐syn and in wild‐type mice at 9 months of age. The initial high absolute values after returning the mice to the cage are caused by stress and were not used for analysis.

Transgenic MBP1‐*α*‐syn showed a trend of reduced ChAT‐positive cells in the nucleus ambiguous compared to wild‐type mice (Fig. 2, *P* = 0.1028). Mean arterial blood pressure responses to trimethaphan (tg: −21 ± 8 vs. wt: −10 ± 5 mmHg, *P* = 0.240) and to clonidine (tg: −8 ± 3 vs. wt: −5 ± 2 mmHg, *P* = 0.314) were similar in both strains. HR responses to trimethaphan (tg: −90 ± 25 vs. wt: −98 ± 22 beats/min), and to clonidine (tg: −188 ± 21 vs. wt: −163 ± 33 beats/min) did not differ between strains. Figure 3 shows the BP and HR values measured for 1 h immediately after the drug injection during pharmacological testing at 9 months of age. We obtained similar results during pharmacological testing at 9 and 12 months of age. The time courses of BP and HR responses were almost identical in both strains.

**Figure 4.**
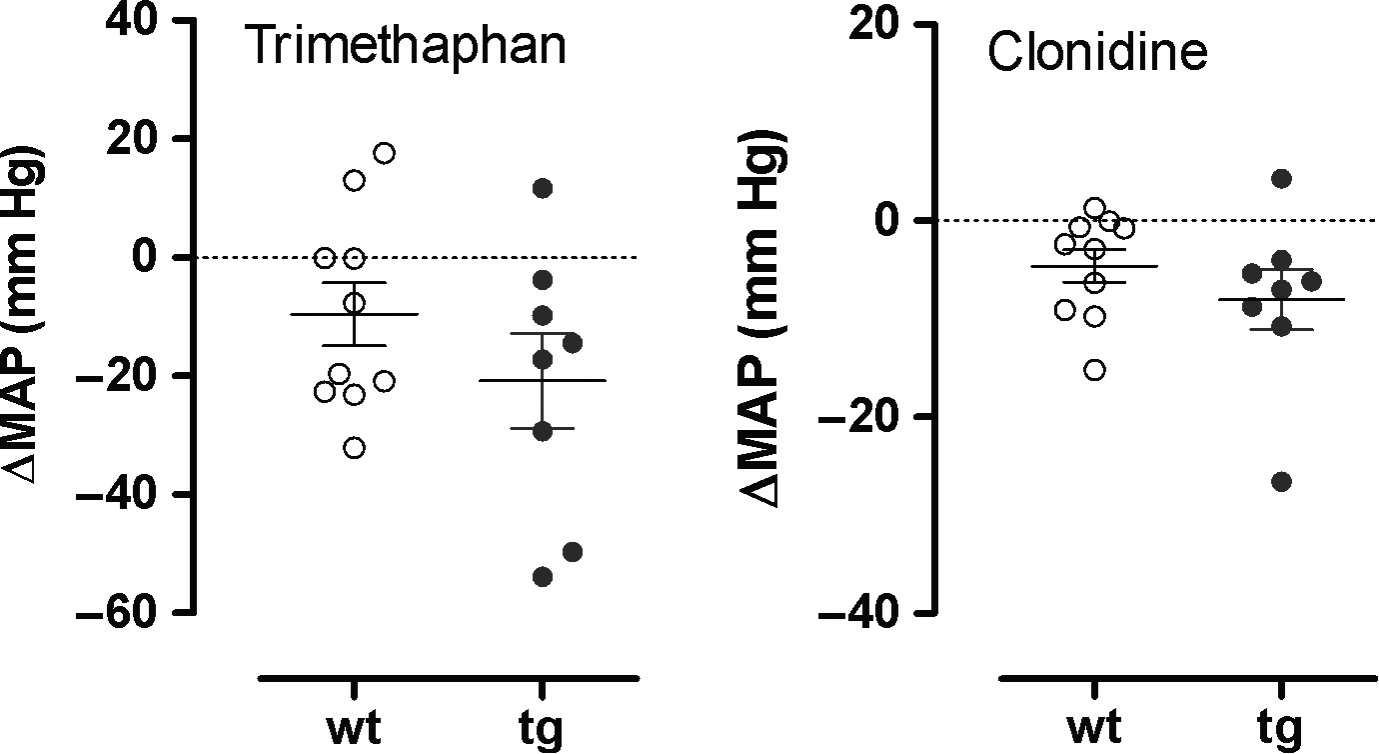
Individual BP changes in wild‐type (wt) and in MBP1‐*α*‐syn (tg) mice in response to trimethaphan (left) and clonidine (right). Changes in BP and HR are expressed as change relative to 1‐h baseline prior to drug administration.

Figure 4 illustrates individual BP responses to trimethaphan and clonidine compared to baseline in transgenic and in wild‐type mice. These responses did not significantly differ between strains. Numerically, transgenic animals tended to have a more pronounced depressor response. However, the difference between groups was rather small and much less than in human MSA.

**Figure 5.**
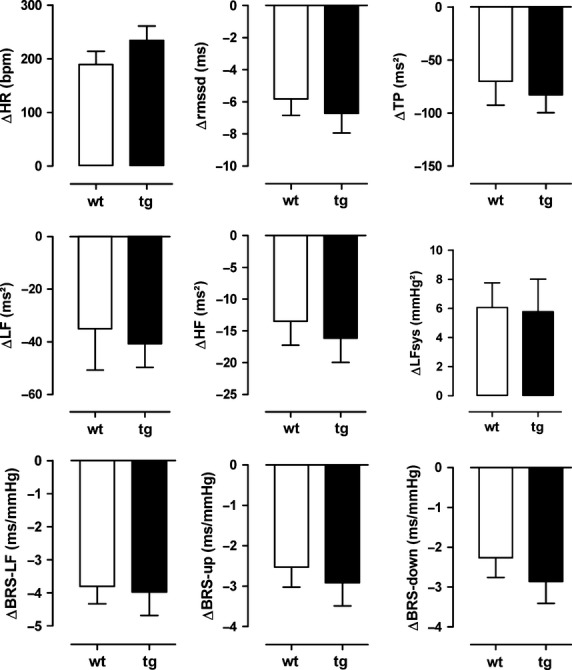
Responses (Δ) of heart rate (HR), heart rate variability in the time domain (rmssd = root mean square of successive differences), heart rate variability in the frequency domain (TP = total power, LF = power in the low‐frequency range, HF = power in the high‐frequency range), systolic blood pressure variability in the low‐frequency range (LFsys) and baroreflex sensitivity calculated by cross‐spectral analysis (BRS‐LF) or by the sequence technique (BRS‐up, BRS‐down) to atropine (2 mg/kg) in MBP1‐*α*‐syn (tg) and in wild‐type (wt) mice at 9 months of age.

Baroreflex sensitivity (BRS‐LF, tg: 4 ± 1 vs. wt: 4 ± 1 msec/mmHg) and HR variability (total power, tg: 84 ± 17 vs. wt: 65 ± 21 msec²) were similar under resting conditions and during pharmacological testing. Figure 5 illustrates changes in HR, HR variability, and baroreflex sensitivity in response to atropine. A similar increase in HR after atropine (tg: +159 ± 24 vs. wt: +146 ± 22 beats/min) was accompanied by similar decreases in HR variability in the time and frequency domains. Moreover, BP variability in the low‐frequency band increased by the same amount in both strains. Baroreflex sensitivity measured with the cross‐spectral method in the low‐frequency range as well as with the sequence technique was reduced by the same amount in both strains. Pharmacological testing at 12 months of age provided similar results.

## Discussion

The main finding of our study is that heart rate and blood pressure regulation in adult transgenic mice overexpressing at moderate levels human alpha‐synuclein in oligodendrocytes is not severely impaired compared to wild‐type mice. In particular, we observed no abnormalities in the diurnal blood pressure profile or heart rate and blood pressure responses to pharmacological testing at 9 and 12 months of age. Finally, heart rate variability, blood pressure variability, and baroreflex sensitivity were also intact in transgenic animals.

Use of a well‐characterized transgenic mouse model expressing human alpha‐synuclein under the control of the murine myelin basic protein promoter is a strength of our study (Shults et al. 2005). Several lines expressing low, intermediate, and high levels of alpha‐synuclein were developed. Expression of moderate levels of alpha‐synuclein (line 1) under the murine myelin basic protein promoter resulted in the formation of aggregates in oligodendrocytes in cortical and subcortical regions and was associated with axonal loss and mild behavioral motor deficits. Mice expressing high alpha‐synuclein levels (line 29) exhibited severe neurological symptoms including tremors, ataxia, and seizure activity and died prematurely (Shults et al. 2005). In similar recently developed transgenic animal models, alpha‐synuclein expression in oligodendrocytes was driven by the 2,’ 3′‐cyclic nucleotide 3′‐phosphodiesterase (CNP) (Yazawa et al. 2005) or the proteolipid protein promoter (PLP) (Kahle et al. 2002). Neurodegeneration with alpha‐synuclein overexpression in oligodendrocytes included neural circuits governing autonomic cardiovascular responses (Kahle et al. 2002). Alpha‐synuclein accumulation, neurodegeneration, and neurological symptoms appear to be particularly pronounced when alpha‐synuclein expression is driven by the murine myelin basic protein promoter (Shults et al. 2005). Thus, our model was suitable assessing influences of alpha‐synuclein in oligodendrocytes on autonomic cardiovascular regulation. We chose to analyze MBP1‐*α*‐syn line 1 mice because they overexpress synuclein at moderate levels and the mice have a normal life expand in contrast to higher expresser lines that die prematurely precluding adequate assessment of cardiovascular regulation. Another strength of our study is the comprehensive assessment of cardiovascular autonomic function using physiological and pharmacological methodologies over several months. Studies using radiotelemetry monitoring in mice over several months are rare. Long‐term monitoring in older mice allows to detect and to follow cardiovascular changes secondary to neurodegenerative processes over time.

We expected MBP1‐*α*‐syn mice to model cardiovascular autonomic abnormalities typically observed in MSA patients with autonomic failure. While orthostatic hypotension cannot be assessed in mice, other MSA‐associated blood pressure abnormalities can be ascertained. Food ingestion and exercise induce vasodilation in splanchnic tract and working musculature, respectively. Because baroreflex‐mediated increases in sympathetic activity cannot compensate for the vasodilation in MSA patients, blood pressure decreases profoundly (Sharabi et al. 2006). Since mice are physically active and fed during the night, the blood pressure profile should be profoundly altered in MBP1‐*α*‐syn mice. Yet, wild‐type and MBP1‐*α*‐syn mice showed normal blood pressure increases during the active phase and reductions during the resting phase. Due to loss of baroreflex restraint, residual sympathetic activity drives an increase in supine blood pressure in many MSA patients. We did not observe an increase in blood pressure in MBP1‐*α*‐syn mice. Furthermore, the depressor response to ganglionic blockade or clonidine was not excessive. Together with normal blood pressure variability, heart rate variability, and baroreflex sensitivity, our study excludes that sympathetic efferents are disconnected from central nervous input in MBP1‐*α*‐syn mice with moderate overexpression of human alpha‐synuclein in oligodendrocytes.

The most likely explanation for our findings is that the level of alpha‐synuclein overexpression may not have been sufficient to promote degeneration of subcortical structures involved in regulation of cardiovascular function. The regulation of such functions depends on the rostral ventrolateral medulla sending glutamatergic projections to spinal sympathetic preganglionic neurons located in the intermediolateral nucleus of the spinal cord. Hence, when baroreceptors are activated through increased blood pressure, the baroreflex afferents in the nucleus tractus solitarius (NTS) activate the caudal ventrolateral medulla neurons (CVLM), which in turn restrain efferent sympathetic activity generated in the rostral ventrolateral medulla (RVLM), thus lowering blood pressure. Moreover, these mice might represent early stages of MSA prior to the development of overt degenerative pathology. Yet, the observation that parasympathetic heart rate regulation is altered in transgenic mice overexpressing human alpha‐synuclein under the control of the PLP promoter as well as the impaired HR responses to BP lowering by nitroprusside (Fleming et al. 2013) in MBP1‐*α*‐syn mice support the idea that alpha‐synuclein accumulation can affect cardiovascular autonomic regulation (Kuzdas et al. 2013). The trend of reduced ChAT‐positive cells in the nucleus ambiguous, the origin of vagal preganglionic neurons projecting directly to the ganglia near the heart, may support this hypothesis. A lower number of ChAT‐positive cells may cause a reduced vagal tone and a lesser response to atropine. However, the reduction in ChAT‐positive cells was not sufficient to cause differences in the response to atropine in our study. MBP1‐*α*‐syn mice express lower levels of synuclein compared to the PLP tg mice. Mice driven by MBP promoter at higher levels such as line MBP29 might display more pronounced loss of ChAT‐positive cells and related deficits, this will be the focus of a follow‐up study.

Another possible explanation for the absence of severe sympathetic dysfunction in MBP1‐*α*‐syn mice is that mice, in general, are difficult models for human autonomic disease. Possibly, in murine model, subcortical cells and RVLM are resistant to degeneration. In addition, different promoters may target different subtypes of oligodendrocytes and may drive alpha‐synuclein only in a subset of oligodendroglial cells. Therefore, a model that is most relevant to human degenerative patterns should be selected for future studies, in order to develop effective new therapeutic strategies.

We previously observed that in diabetic NOD mice, diabetes produces sympathetic dysfunction while cardiac vagal control and baroreflex sensitivity were preserved (Gross et al. 2008). Diabetic patients exhibit opposite changes in cardiovascular autonomic regulation with vagal dysfunction preceding sympathetic dysfunction. Yet, cardiovascular abnormalities in mice with isolated adrenergic dysfunction due to genetic dopamine‐beta‐hydroxylase deletion resemble clinical abnormalities in patients (Usera et al. 2004). Given the limited contribution of vagal tone to resting cardiovascular regulation in mice compared to human subjects, abnormalities in sympathetic cardiovascular control may be easier to detect in mouse models. Sympathetic dysfunction was the expected abnormality in our study. Finally another possibility is that time points beyond 9–12 months may be required to trigger autonomic cardiovascular alterations.

We conclude that adult mice expressing moderate levels of human alpha‐synuclein under the control of the murine myelin basic protein promoter display preserved cardiovascular sympathetic functioning. The observation may suggest that moderate levels of alpha‐synuclein overexpression in oligodendrocytes may not be sufficient to produce damage to subcortical structures involved in cardiovascular regulation and that either higher levels of synuclein or more advanced age is necessary. Indeed, in mice, neurodegeneration is more pronounced when alpha‐synuclein overexpression is combined with deletion of neuroprotective factors (Ubhi et al. 2010). Our findings may be relevant for the development of animal models testing candidate drugs for MSA‐associated sympathetic dysfunction. Furthermore, our findings could provide an impetus not to restrict the search for these drugs to compounds interfering with alpha‐synuclein deposition in oligodendrocytes.

## Conflict of Interest

None declared.
